# Longitudinal Treatment Outcomes of Microsurgical Treatment of Neurosensory Deficit after Lower Third Molar Surgery: A Prospective Case Series

**DOI:** 10.1371/journal.pone.0150149

**Published:** 2016-03-04

**Authors:** Yiu Yan Leung, Lim Kwong Cheung

**Affiliations:** Oral and Maxillofacial Surgery, Faculty of Dentistry, The University of Hong Kong, Hong Kong, Special Administrative Region, The People Republic of China; Navoadaya Dental College and Hospital, mantralayam Road, INDIA

## Abstract

**Objective:**

To prospectively evaluate the longitudinal subjective and objective outcomes of the microsurgical treatment of lingual nerve (LN) and inferior alveolar nerve (IAN) injury after third molar surgery.

**Materials and Methods:**

A 1-year longitudinal observational study was conducted on patients who received LN or IAN repair after third molar surgery-induced nerve injury. Subjective assessments (“numbness”, “hyperaesthesia”, “pain”, “taste disturbance”, “speech” and “social life impact”) and objective assessments (light touch threshold, two-point discrimination, pain threshold, and taste discrimination) were recorded.

**Results:**

12 patients (10 females) with 10 LN and 2 IAN repairs were recruited. The subjective outcomes at post-operative 12 months for LN and IAN repair were improved. “Pain” and “hyperaesthesia” were most drastically improved. Light touch threshold improved from 44.7g to 1.2g for LN repair and 2g to 0.5g for IAN repair.

**Conclusion:**

Microsurgical treatment of moderate to severe LN injury after lower third molar surgery offered significant subjective and objective sensory improvements. 100% FSR was achieved at post-operative 6 months.

## Introduction

Trigeminal nerve injury is a notorious complication of lower third molar surgery. Permanent lingual nerve (LN) or inferior alveolar nerve (IAN) deficits were shown to affect quality of life of the affected individuals [1,2]. A study from our center has shown patients with trigeminal neurosensory deficits might have more depressive symptoms and worse life satisfaction that are comparable to those with severe neurological deficit like spinal cord injury [3]. The social life and mood of these patients might be drastically affected by the symptoms of the nerve injury like dysaesthesia or hyperaesthesia, who would then be very desperate to seek treatment for these disturbing conditions.

A systematic review of treatment modalities on trigeminal nerve injury as a consequence of lower third molar surgery have shown microsurgical repair is the mainstream treatment and is well studied in the literature [4]. This treatment modality was reported to be able to bring sensory improvement to variable extents, yet complete recovery was rare. However, most studies that were included in this systematic review were retrospective in nature. The reported outcomes were mostly cross-sectional and the methods of neurosensory assessment were not standardized in these studies. The post-operative longitudinal change of sensation from the patient’s perspective and the objective neurosensory outcomes remain unclear.

The aim of the study was to prospectively evaluate the longitudinal subjective and objective treatment outcomes of the microsurgical treatment of LN and IAN injury after lower third molar surgery.

## Materials and Methods

This was a prospective longitudinal observational study to investigate the surgical treatment outcomes of LN or IAN injury as a consequence of lower third molar surgery. Ethical approval has been obtained from the Institutional Review Board of The University of Hong Kong and Hong Kong West Cluster. Patients with LN or IAN injury referred to the Discipline of Oral and Maxillofacial Surgery, Faculty of Dentistry, The University of Hong Kong for management were assessed for recruitment eligibility by the following inclusion and exclusion criteria:

**Inclusion criteria:**
- Patients with post-operative moderate to severe LN or IAN injury presenting with one or more of the following symptoms:
  1. Moderate to severe hypoaesthesia or anaesthesia (with subjective Numbness Visualized Analog Scale (VAS) [0 (normal) -10 (most severe)] scoring 5 or above (see “The Neurosensory Tests” section);
  2. Dysaesthesia (pain at the supplying area of the associated nerve);
  3. Taste disturbance in LN injury
- The neurosensory deficit was a consequence of lower third molar surgery.
- The patient agreed to undergo microsurgical treatment of the injured LN or IAN under general anaesthesia.
  **Exclusion criteria:**
  1. Patients with pathologies affecting the neurosensory function of LN and IAN
  2. Patients underwent any maxillofacial surgery (e.g. orthognathic surgery), other than the lower third molar surgery, that caused LN or IAN neurosensory deficit.
  3. Patients with systemic condition predisposing to local infection: diabetes, acquired immune deficiency syndrome (AIDS), on chemotherapy or immunosuppressive therapy, head and neck cancer
  4. Patients with history of radiotherapy to the head and neck region

Eligible patients who were recruited had provided written informed consent to participate in this study. An informed consent of the surgical procedures and risks including the possibility of worsening of sensation was explained and signed by each patient. The patients were then scheduled for the earliest available time for the microsurgical treatment of the injured LN or IAN under general anaesthesia in Queen Mary Hospital, Hong Kong.

The following data were recorded preoperatively:

1. Age (at the time of microsurgery of the nerve) and gender of the patient.
2. The side of the lower third molar surgery that caused the neurosensory deficit
3. The nerve involved.
4. The time lapse from the nerve injury to the date of the microsurgery.
5. Results of the subjective and objective neurosensory tests (Refer to the section The Neurosensory Tests)

Postoperatively the subjects were reviewed at 1^st^ week, 1^st^, 3^rd^, 6^th^ and 12^th^ month. The subjective and objective neurosensory tests were performed in each of these post-operative follow-up appointments.

### The Neurosensory Tests

To assess the subjective perception of the patients and to provide an objective assessment of the neurosensory condition, a set of standardized neurosensory tests were used.

#### Subjective assessments

The following parameters were rated by the subjects on a visualized analog scale (VAS) from 0 (normal) to 10 (most severely affected):

- Numbness
- Hyperaesthesia
- Pain
- Taste disturbance
- Speech
- Social life impact

#### Objective assessments

Three tests were carried out for both LN and IAN deficit subjects to compare the affected side and non-affected side. Taste sensations were tested only for LN deficit subjects. The objective tests were as follows:

Light Touch Threshold: Von Frey monofilament fibres of ascending diameter were applied to the tongue / lip and the lightest fibre (in terms of gram of force to bend the fibre) a subject could feel were recorded. When the tip of a fibre of given length and diameter was pressed against the skin / mucosa at right angle, the force of application increased as long as the researcher continues to advance the probe, until the fibre bends. The set consisted of 20 fibres of different diameters. It was recorded as “could not sense” if the subject could not feel the largest fibre.

Two-point discrimination: A decagonal acrylic disc containing of 10 sets of paired blunt metallic probes of 0.8mm diameter with separations ranging from 2-20mm at 2mm interval were applied with a constant force to the tongue or lip at an ascending order. The smallest separations of the probes that a subject could discriminate a two-point sensation were recorded. It was recorded as “could not sense” if the subject could not feel the biggest separation of 20mm.

Pain threshold: A blunted 19G needle connected to a spring gauge were applied to the tongue or lower lip until the subject started to feel “pain” as the force gradually increased and the force in terms of gram were recorded. Three readings were taken and the mean value was recorded. It was recorded as “could not sense” if the subject could not feel pain larger than 100 gram.

Taste sensation: Cotton wool pellets soaked in solution composed of either 4M sodium chloride, 0.1M quinine, 5% acetic acid or 3M sucrose, corresponding to salt, bitter, sour or sweet taste, respectively, were applied to the dorsal tongue surface in a double blinded (patient and researcher blind) randomized order. Only the nurse who handed the cotton wool pellets to the examiner knew which solution. The subject was asked by the examiner which taste he/she was tasting and the nurse, who was knew the test medium used, would mark whether the answer was correct, incorrect or no taste was sensed. The tested area was cleaned before another solution soaked with cotton pellet was applied.

### Surgical Techniques

The microsurgical treatment techniques of the LN and IAN used is described in this section.

**Fig 1:**
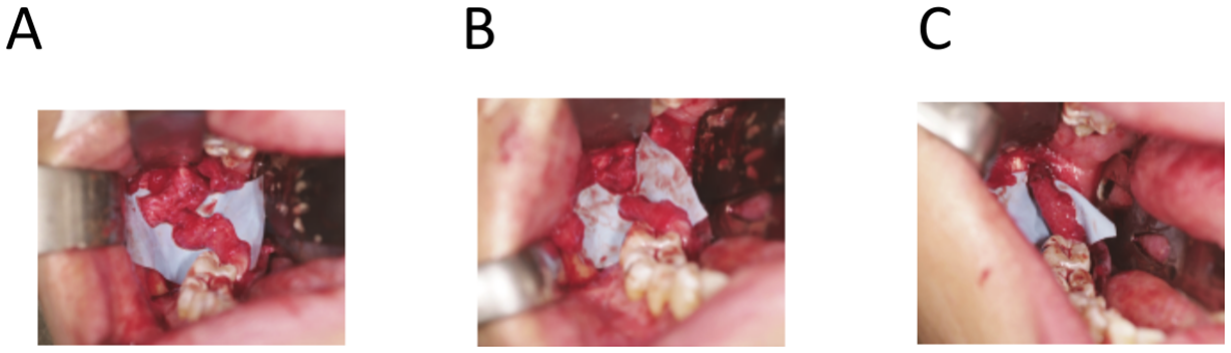
Microsurgical exploration and repair of lingual nerve. (a) Traumatized lingual nerve was exposed showing the traumatic neuroma-in-continuity. (b) Proximal and distal nerve stumps prepared for anastomosis. (c) Anastomosis of the lingual nerve by epineural suturing.

A. Microsurgical exploration and repair of LN (Fig 1)

A buccal incision was made along the external oblique ridge to the distobuccal margin of lower second molar. A separate incision was made along the lingual gingival margin from the lower second molar to the ipsilateral canine to allow sufficient exposure. The buccal flap and lingual flap connecting at the distal part of the lower second molar were raised with periosteal elevators. The lingual periosteum were then be incised. With careful blunt dissection, the lingual nerve were exposed and explored. External neurolysis were performed if the nerve was adhered to the adjacent tissue. Any traumatic neuroma was excised, measured and sent for histopathology evaluation. The proximal and distal nerve stumps were mobilized and the ends were prepared for anastomosis. The ends should meet without tension. Epineural suturing with four to six 7/0 nylon sutures were performed. The wound was closed with 4/0 polyglactin (Vicryl) sutures after haemostasis and final inspection of the integrity of nerve suturing.

**Fig 2:**
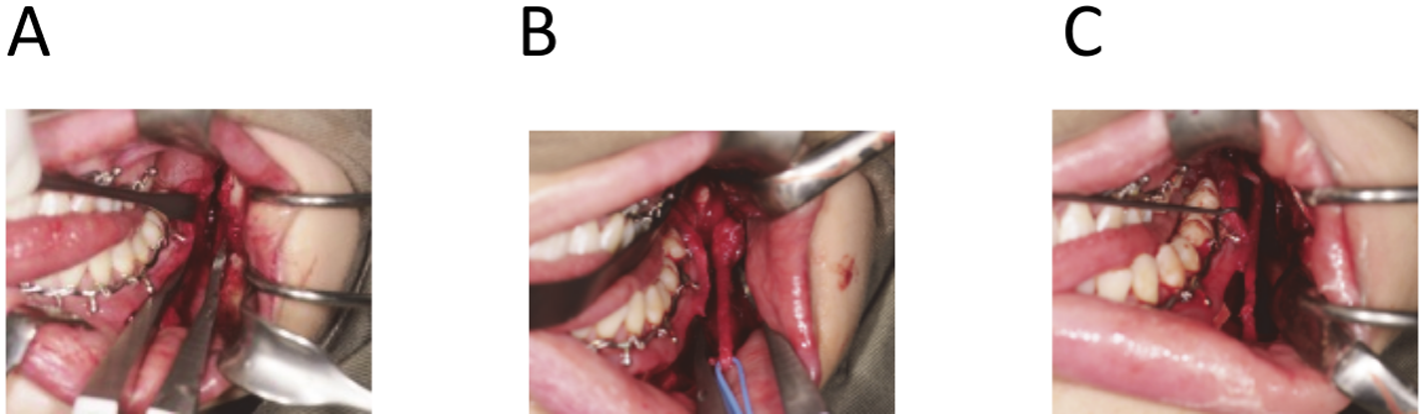
Microsurgical exploration and repair of inferior alveolar nerve. (a) Sagittal split of the mandible was performed after all the cuts were made. (b) The nerve was exposed showing the traumatic neuroma-in-continuity. (c) Anastomosis of the inferior alveolar nerve by epineural suturing.

B. Microsurgical exploration and repair of IAN (Fig 2)

An extended sagittal split osteotomy approach was used. An incision was made along the buccal vestibule from the retromolar region to the canine region. The mucoperiosteal flap was raised to expose the lingula up to the sigmoid notch on the medial aspect of the ramus and on the lateral aspect of the body of mandible reaching the mental foramen. A bucco-vertical cortical cut was made at 1cm distal to the mental foramen by a fine Lindermann bur. A four-hole titanium mini-plate was adapted across the osteotomy cut. Four holes were drilled and the plate was removed. The medio-horizontal osteotomy cut was performed on the medial ramus above the lingual level and at approximately 5mm below the sigmoid notch, bisecting the upper and lower occlusal plane when the mouth was opened wide. The bucco-vertical osteotomy and the medio-horizontal osteotomy cuts were joined with an osteotomy cut along the external oblique ridge. The mandible was splitted by a pair of blunt osteotomes. The course of the IAN was traced and freed from its bony canal. Any neuroma was dissected and the proximal and distal neural ends were then exposed. Traumatic neuroma if present were excised, measured and sent for histopathology assessment. The IAN was freed from the mental foramen by cortical bone removal extending forward from the bucco-vertical cut to reach 5mm anterior from the mental foramen. The ends were mobilized until the ends could meet without tension. The proximal and distal nerve stumps were trimmed. Epineural suturing with four to six 7/0 nylon sutures were performed. The original occlusion was re-established and the buccal bony segment was fixed with the prepared mini-plate by four mini-screws on the pre-drilled holes. The wound was closed with 4/0 polyglactin (Vicryl) sutures after haemostasis

All the operations were performed under general anaesthesia. A dose of antibiotics and dexamethasone were given intravenously at induction of anaesthesia. Post-operatively each patient was prescribed with sufficient analgesics, a 2-day intravenous course of antibiotics followed by 5 days of oral antibiotics. Dexamethasone 4mg were given twice a day for 2 days to reduce post-surgical oedema of the IAN.

### Treatment Outcomes

The subjects’ demographic data, time lapse of injury to treatment and subjective and objective neurosensory data were collected before surgery. The postoperative recovery patterns after microsurgical treatment of LN or IAN were presented in a longitudinal manner. The subjective and objective neurosensory outcomes at post-operative 12 months were compared to the pre-operative data. Functional Sensory Recovery (FSR) was considered achieved after treatment if the objective tests showing static 2-point discrimination less than 20mm and superficial pain / tactile sensation without over-reaction.

## Results

There were 12 subjects (10 female) recruited in this study from September 2009 to December 2012. The mean age was 28.3 years (S.D. 5.0 years), with age ranged from 22 years to 39 years. 16.7% (2/12) of the subjects had IAN injury and 83.3% (10/12) had LN injury. The mean time lapse of nerve injury to microsurgery was 6.3 months (S.D. 4.0 months), which was ranged from 1 month to 14 months. 10 subjects (83.3%) had exploration and repair of LN and 1 subject (8.3%) had exploration and repair of IAN. One subject (8.3%) had exploration of IAN and did not found any neuroma formation, which the IAN was freed from the scar tissue of adjacent structure (external neurolysis) was performed. The mean length of the excised neuroma from other 11 cases was 10.2mm (S.D. 5.5mm), which was ranged 6mm to 22mm. There were 41.6% (5/12) of the subjects taken medico-legal action against the dental practitioners who removed the lower third molar that caused the nerve injury. The data is presented in Table 1.

**Table 1:** Demographic and surgical-related data of the included subjects.

**Gender**	
Male	17% (2/12)
Female	83% (10/12)
**Mean Age (S.D.)**	28.3 years (5.0 years)
**Tooth involved**	
Lower left third molar	33.3% (4/12)
Lower right third molar	66.7% (8/12)
**Nerve involved**	
LN	83% (10/12)
IAN	17% (2/12)
**Time lapse from injury to surgery (S.D.)**	6.3 months (4.0 months)
**Surgical Procedures**	
Exploration and repair of LN	83% (10/12)
Exploration and repair of IAN	8.3% (1/12)
Exploration and external neurolysis of IAN	8.3% (1/12)
**Mean length of neuroma (S.D.)**	10.2mm (5.5mm)
**Medico-legal action**	41.7% (5/12)

### Subjective outcomes of microsurgery after lingual nerve injury

**Fig 3:**
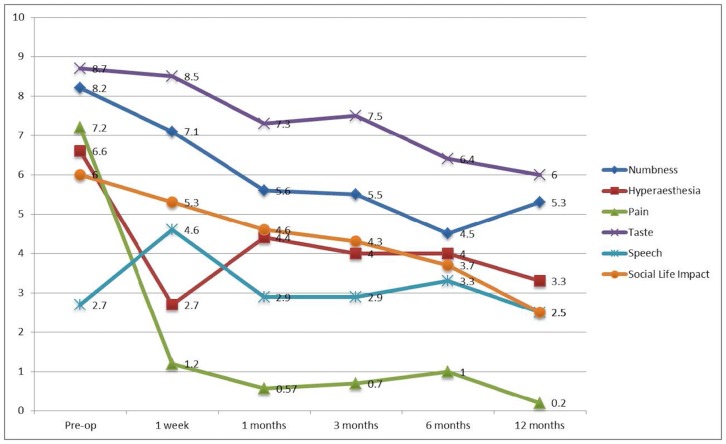
Change of subjective outcomes after microsurgery of lingual nerve (n = 10).

The subjective outcomes after exploration and repair of LN were presented in Fig 3. It was noted the mean VAS of numbness has improved from 8.2 pre-operatively to 5.3 at post-operative 12 months. There were 7 subjects presented with hyperaesthesia of the affected area with a mean VAS 6.6 pre-operatively. It was noted the mean hyperaesthesia VAS dropped to 2.7 at post-operative 1 week, which subsequently returned to 4.4 at post-operative 1 month and gradually reduced further to 3.3 at post-operative 12 months. The mean VAS for pain, taste and social life impact dropped continuously from pre-operative 7.2, 8.2 and 6 to 0.2, 6 and 2.5 at post-operative 12 months, respectively. The mean VAS for speech was noted to get worse at post-operative 1 week from 2.7 to 4.6 but gradually reduced to 2.5 at post-operative 12 months.

The outcomes of the various subjective parameters at post-operative 12 months were categorized to “Recovered” (VAS = 0 at post-operative 12 months), “Improved” (VAS at post-operative 12 months lower than pre-operative), “Same” (VAS at post-operative 12 months same as pre-operative) or “Deteriorated” (VAS at post-operative 12 months higher than pre-operative) (Table 2). It was noted 90% of the subjects (9/10) presented with numbness pre-operatively showed improvement at post-operative 12 months, with 10% (1/10) complained of deterioration of numbness sensation. 28.6% (2/7) of the subjects with hyperaesthesia recovered, with 14.3% (1/7) showed some improvement and 28.6% (2/7) felt worsened hyperaesthesia. 75% (3/4) of the subjects recovered from pain symptoms with 25% (1/4) had worsened pain symptoms. 70% (7/10) of the subjects had improvement of recovery of subjective taste sensation. Half of the subjects had recovery or improvement on speech but the other half subjectively felt the speech was worse at post-operative 12 months. Most (90%) subjects felt the overall social life impact was improved or recovered at post-operative 12 months.

**Table 2:** Subjective outcomes after microsurgery of LN and IAN at post-operative 12 months.

	Lingual Nerve (n = 10)	Inferior Alveolar Nerve (n = 2)
**Numbness**		
Pre-op with numbness	n = 10	n = 2
Post-op 12 month		
Improved	90% (9/10)	100% (2/2)
Deteriorated	10% (1/10)	0
**Hyperaesthesia**		
Pre-op with hyperaesthesia	n = 7	n = 1
Post-op 12 month		
Recovered	28.6% (2/7)	
Improved	14.3% (1/7)	100% (1/1)
Same	28.6% (2/7)	
Deteriorated	28.6% (2/7)	
**Pain**		
Pre-op with pain	n = 4	n = 2
Post-op 12 month		
Recovered	75% (3/4)	100% (2/2)
Deteriorated	25% (1/4)	0
**Taste**		
Pre-op with taste disturbance	n = 10	Not applicable
Post-op 12 month		
Recovered	10% (1/10)	
Improved	60% (6/10)	
Same	20% (2/10)	
Deteriorated	10% (1/10)	
**Speech**		
Pre-op with speech affected	n = 8	n = 0
Post-op 12 month		
Recovered	12.5% (1/8)	
Improved	37.5% (3/8)	
Deteriorated	50% (4/8)	
**Social Life**		
Pre-op with social life impact	n = 10	n = 2
Post-op 12 month		
Recovered	30% (3/10)	0
Improved	60% (6/10)	50% (1/2)
Same	0	50% (1/2)
Deteriorated	10% (1/10)	0

### Objective outcomes of microsurgery after lingual nerve injury (Table 3)

**Table 3:** Change of objective outcomes after microsurgery of lingual nerve (n = 10) and inferior alveolar nerve (n = 2).

	Unaffected side	Pre-op	1 week	1 month	3 month	6 month	12 month
**Static Light Touch (S.D.) (gram)**							
Lingual nerve	0.029 (0.015)	44.7 (92.7)	11.2 (18.7)	2.1 (2.1)	1.6 (1.9)	0.73 (1.4)	1.2 (2.1)
Inferior alveolar nerve	0.024 (0.023)	2.0 (2.8)	154.0 (206.0)	15.0 (0)	1.5 (0.71)	0.90 (0.71)	0.50 (0.14)
**2-point Discrimination (mm)**							
Lingual nerve	5.6 (2.3)	13.2 (3.3)^a^	12.0 (6.8)^b^	10.3 (5.5)	9.0 (4.8)	10.2 (5.2)	7.4 (5.4)
Inferior alveolar nerve	6.0 (0)	14.0 (0)^c^	15.0 (1.4)	12.0 (0)	14.0 (2.8)	11.0 (1.4)	16.0 (0)
**Pain Threshold (gram)**							
Lingual nerve	28.4 (6.5)	54.2 (20.0)^d^	52.6 (23.0)^e^	57.0 (22.6)^f^	39.6 (14.5)^g^	41.8 (19.4)	43.7 (19.6)
Inferior alveolar nerve	31.2 (15.8)	55.6 (0)^h^	42.0 (0)^i^	82.0 (0)	59.0 (41.0)	46.0 (8.5)	42.6 (17.0)

^a^ 5 subjects could not detect 2 points at the maximal distance (i.e. >20mm)

^b^ 3 subjects could not detect 2 points at the maximal distance (i.e. >20mm)

^c^ 1 subject could not detect 2 points at the maximal distance (i.e. >20mm)

^d^ 4 subject could not feel pain at the maximal force (i.e. >100g)

^e^ 4 subject could not feel pain at the maximal force (i.e. >100g)

^f^ 2 subject could not feel pain at the maximal force (i.e. >100g)

^g^ 1 subject could not feel pain at the maximal force (i.e. >100g)

^h^ 1 subject could not feel pain at the maximal force (i.e. >100g)

^i^ 1 subject could not feel pain at the maximal force (i.e. >100g)

There were improvements in all three objective neurosensory parameters. The mean detectable force in gram for static light touch improved from 44.7g pre-operatively to 1.2g at post-operative 12 months, but was greater than the unaffected side (0.029g). Five of the ten subjects could not detect 2 points pre-operatively but all could detect 2 points on the 2-point discrimination test at post-operative 12 months. The mean distance for 2-point discrimination was improved from 13.2mm pre-operatively (for those who detected 2 points) to 7.4mm at post-operative 12 months. For pain threshold test, pre-operatively 4 subjects could not feel pain at the maximal force of over 100g, with a mean force in gram of 54.2g for those who could feel pain at the affected site. At post-operative 12 months, all subjects could feel pain and the mean force in gram for pain threshold was improved to 43.7g. The outcomes of the various objective parameters at post-operative 12 months were categorized to “Recovered” (same as the unaffected side), “Improved” (more sensitive than pre-operative tests), “Same” (same sensitivity to pre-operative test) and “Deteriorated” (worse when compared to pre-operative tests) (Table 4). At post-operative 12 months, 90% of the subjects had improved or recovered static light touch and 2-point discriminations when compared to the preoperative status. Pain threshold was recovered in 40% of the subjects. There were 70% of the subjects who had objective taste improvement but none of them had recovered all four taste sensations. Of note, 10% (1/10) of the subjects had deteriorated objective sensation at 12 months post-operatively.

**Table 4:** Objective outcomes after microsurgery of LN and IAN at post-operative 12 months.

	Lingual Nerve (n = 10)	Inferior Alveolar Nerve (n = 2)
**Static Light Touch**		
Recovered	10% (10/10)	0
Improved	80% (8/10)	50% (1/2)
Deteriorated	10% (1/10)	50% (1/2)
**2-point Discrimination**		
Recovered	20% (2/10)	0
Improved	70% (7/10)	100% (2/2)
Deteriorated	10% (1/10)	0
**Pain Threshold**		
Recovered *	40% (4/10)	0
Improved	30% (3/10)	100% (2/2)
Same	20% (2/10)	0
Deteriorated	10% (1/10)	0
**Taste**		Not applicable
Improved	70% (7/10)	
Same	10% (1/10)	
Deteriorated	20% (2/10)	

* Pain threshold <5gram compared to the unaffected side.

### Subjective outcomes of microsurgery after inferior alveolar nerve injury

**Fig 4:**
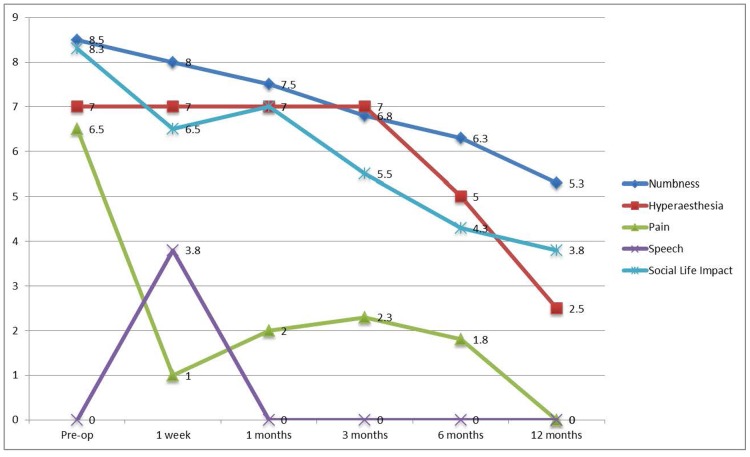
Change of subjective outcomes after microsurgery of inferior alveolar nerve (n = 2).

The subjective outcomes after microsurgery of IAN were presented in Fig 4. The mean numbness VAS of the two subjects who underwent microsurgery of IAN had improved gradually from pre-operative 8.5 to 5.3 at post-operative 12 months. One subject with hyperaesthesia had a VAS of 7 pre-operatively. The hyperaesthesia maintained at the same level post-operatively until post-operative 6 months which then showed gradual improvement to 2.5 at post-operative 12 months. Both subjects complained pain with a mean VAS of 6.5 pre-operatively, which was improved after microsurgery and were both recovered at 12 months post-operatively. The microsurgery had affected the speech of both patients temporarily at post-operative 1 week and recovered afterwards. There was also a gradual improvement in terms of social life impact from VAS 8.3 pre-operatively to 3.8 at post-operative 12 months.

For the categorized subjective outcome as shown in Table 2, it was noted at post-operative 12 months there were improvements of numbness and hyperaesthesia in the subjects. The pain symptom was recovered in both subjects which was presented pre-operatively. One subjects felt the impact on social life was improved but the other felt it maintained the same.

### Objective outcomes of microsurgery after inferior alveolar nerve injury (Table 3)

The mean force was 2.0g pre-operatively for the static light touch. It was noted the static light touch sensation reduced to 154g at the first post-operative week, and showed gradual improvement at post-operative 12 months, which was improved but remained higher than the unaffected side (0.024g). One of the two subjects could not detect 2 points pre-operatively but was able to sense it at post-operative 12 months. The mean distance of 2-point discrimination remained much insensitive when compared to the unaffected side (16mm versus 6mm). One of the two subjects could not feel pain at 100g pre-operatively but was able to sense it at post-operative 12 months. The pain threshold was 46g at post-operative 12 months, which was improved when compared to pre-operative reading but remained less sensitive than the unaffected side.

One subject had improvement and the other had deterioration in the static light touch at post-operative 12 months when compared to pre-operative measurement. Both subjects presented with improved 2-point discrimination and pain threshold when compared to the pre-operative measurements. None of the subjects had full recovery in any of the three objective tests matching the contralateral normal side (Table 4).

### Functional Sensory Recovery

It was noted that all subjects for LN and IAN repair had static 2-point discrimination less than 20mm and superficial pain / tactile sensation without over-reaction. FSR was achieved in all subjects for LN and IAN repair by 6 months post-operatively.

## Discussion

The key findings of this prospective longitudinal study were most patients who received microsurgery as a treatment of LN or IAN injury after lower third molar surgery had subjective and objective sensory improvement. Perception of pain as a consequence of LN or IAN injury had the highest proportion of complete recovery after treatment. The majority of the patients reported subjective perceptions including numbness, hyperaesthesia, taste (in LN injury) and social life compromise had improvement after treatment but were not completely recovered at the time of 12 months post-operatively. Objective quantitative sensory tests including static light touch, 2-point discrimination, pain threshold and taste (in LN injury) also showed improvement in most patients after treatment.

Neurosensory deficit after lower third molar surgery is a potentially serious complication. It has been shown in previous studies that it can significantly affects an individual’s general and oral health-related quality of life as well as developing more depressive symptoms and affects life satisfaction [1–3]. It was not surprising to note that 41.7% of the subjects undertook medico-legal action against the dentists who caused the nerve injury. Microsurgical nerve repair has remained the most popular treatment for severe trigeminal nerve injury, and its outcome has been reported in several studies [5–13]. It has been reported that recovery might occur in around 60% of patients who had LN or IAN deficit after third molar surgery in general [14]. However, when the nerve injury is severe (axonotmesis or neurotmesis), the prognosis of spontaneous recovery is expected to be poor. Severe LN or IAN injury may be presented as persistent anaesthesia or severe hypoaesthesia without sign of recovery in the early post-operative period or taste disturbance in LN injury [5]. Traumatic neuroma may be formed and may contribute to the development of disturbing symptoms like hyperaesthesia or spontaneous pain. From our Discipline’s experience, Hong Kong Chinese in general were quite reluctant to receive surgical treatment, which may be related to the traditional Chinese culture of preferring non-surgical methods instead. Hence, a patient would seek surgical treatment for the LN or IAN deficit only when the symptoms were very severe and disturbing his/her social life, or in another words, there was “nothing to lose” to receive the surgical treatment. This has likely accounted for the smaller sample size in this study. It was also noted there were more patients had surgical treatment for LN injury than IAN injury. This finding concurs with the systematic review of from our center that found most studies on surgical nerve repair were on lingual nerve [4]. It might be explained by the fact that severe LN injury could affect taste sensation, which is an additional negative attribute on the impact of social life on the individual and induces a patient to seek surgical treatment of LN nerve injury.

The best timing of microsurgical repair after trigeminal nerve injury has not come to a conclusion despite debates in the literature [4]. The fact of spontaneous neurosensory recovery should not be overlooked and it usually occurs within the first year after injury [14]. However, patients with moderate-to-severe nerve injury were unlikely to recover completely, and the related symptoms like dysaesthesia might pose physical and psychological impact during the waiting period. In this case series, we discussed the pros and cons of nerve repair with the patients and they would decide whether they would undergo the recommended treatment. It was not surprising to note the patients intended to receive early treatment with their degree of nerve injury severity.

Most studies in the literature reported improvement was expected after microsurgical repair of LN and IAN, but complete recovery of neuro-sensation was rare [12, 15–17]. In the systematic review on the nerve injury treatment, it was found that only 5.7% of the subjects completely recovered after direct suturing in microsurgical repair of LN. There were 83.9% of the subjects who had improvement of various extents [4]. The findings of this study concur with those reported in the systematic review, with the majority of the subjects reported subjective and objective improvement in various parameters. However, sensation is subjective, complex and multidimensional. This study has therefore grouped the subjective effects of the nerve injury into 6 categories, including numbness, hyperaesthesia, pain, taste, speech and social life impact. It was found that in general all six aspects showed improvement to a certain extent. Numbness was the most obvious deficit when LN injury occurs, and with microsurgical treatment 90% of the LN group and 100% of the IAN group had improvement. Pain, or dysaesthesia, was usually the motivation driving a patient to seek surgical treatment. Robinson et al. reported in their prospective study on lingual nerve repair, only a small portion of subjects with pain was recovered [12]. Hillerup and Stoltze also reported a similar finding of disappointing outcome on pain resolution [18]. In our series, 75% of the subjects who had LN repair recovered completely from pain, which was contrary to the British and Dutch authors but was in line with the American study [19]. The outcome of pain recovery after IAN repair was excellent in this study. Hyperaesthesia was also largely improved in both LN group and IAN group. There was a drastic reduction of hyperaesthesia in the early post-operative period in the LN group, which could be explained by the surgical excision of the neuroma and re-anastomosis of the nerve leading to a temporary sensory “disconnection”. When re-entering of the endoneural sheath by the distal stump occurred with time, the hyperaesthesia sensation might be restarted but gradually reduced to a lower level.

In those who had LN repair, it was noted taste recovered to a small extent in this study in most of the subjects, with a mean VAS of 8.7 pre-operatively to 6 at post-operatively 12 months. There was only one subject reported complete recovery, despite 60% had improvement to some extent. It was supported by the literature that a relatively low gustatory recovery rate reported in most of the studies on lingual nerve repair [5, 20, 21]. Robinson and Smith hypothesized the poor taste recovery was due to the gustatory fibres being a minority in the lingual nerve and would have a small chance of being guided to an appropriate terminal site since there is no colour or textural difference between the sensory and taste fibres [21]. This would have implication to the surgeons in explaining this risk of taste recovery to a patient during the decision making of choosing LN repair or not.

Speech was noted to be affected in LN injury but was rarely reported in the literature. Although the motor function of tongue directly controls its movement and the overall phonetic function in speech, numbness may give an individual a false impression of the immobility of the tongue. Cruz et al. reported that 30% of the subjects who had induced lingual nerve anaesthesia reported speech disturbance, even though they presented no perceptual changes in sound quality [22].

Social life impact is a direct subjective measurement on a patient’s quality and satisfaction of life. This study found the subjects who had LN or IAN repair developed drastic improvement in terms of social life impact. 60% of the subjects felt the impact of the neurosensory deficit on their social life had improved and an addition of 30% felt they were back to normal in those who had lingual nerve repair. The Harvard group published two studies on patient satisfaction and oral health related quality of life after trigeminal nerve repair and also found more than half of the patients rated their overall satisfaction to be good to excellent [23]. Our study has further fostered the belief that trigeminal nerve repair would improve the overall social life of the patients who suffered from the consequences of severe trigeminal nerve injury after lower third molar surgery.

It is always a challenge to correlate objective and subjective neurosensory tests in nerve repair studies [24]. To avoid confusion of the clinical outcomes, subjective and objective tests were reported separately. This study is the first in the literature reporting the longitudinal changes of the three objective neurosensory tests after microsurgery of trigeminal nerve. The pre-operative objective test readings were much more insensitive than the normal side, and were even worse than the objective data after trigeminal nerve injury published by Renton et al., showing the subjects all suffered from severe LN or IAN injury [25]. There were progressive improvements in most of the objective neurosensory tests after microsurgery of both LN and IAN, but the outcomes had not recovered to the sensory level of the contralateral normal side. To evaluate the treatment outcomes of nerve injuries, the British Medical Research Council had recommended a grading system that was used for peripheral nerve injury but was also recommended to be used in trigeminal nerve repair [26,27]. A “functional sensory recovery” (FSR) was achieved after nerve repair if the objective tests showing static 2-point discrimination less than 20mm and superficial pain / tactile sensation without over-reaction. Susarla et al. reported 75% of the subjects achieved FSR after post-operative 12 months [24], and Bagheri reported 90.5% and 81.7% of the subjects achieved FSR after LN and IAN repair, respectively[15, 28]. In this study, 2-point discrimination less than 20mm was achieved in all the subjects who had microsurgery of LN and IAN. The result of pain threshold also showed all subjects were able to feel pain sensation on the pin prick test. It was an indicator of restoring the protective mechanism of avoiding further tongue or lower lip biting, as many patients complained of this side effect after trigeminal nerve injury and this could significantly affect their social life. It was noted subjects underwent LN and IAN repair had achieved 100% FSR at 6 months post-operatively.

It should be reminded that in this study, 10% of the patients complained of deterioration of the sensation after surgical repair. It is important for the surgeons and the patients to reach a mutual understanding on the risk of sensory deterioration prior to the surgical treatment. This is also an implication in the informed consent for the clear explanation of such risk.

The strength of this study was the detailed prospective investigations of longitudinal changes after trigeminal nerve repair. The recovery was reported in both objective measureable methods as well as from the patients’ subjective perceptions. The weakness of this study, however, was the relatively small sample size, especially those who received IAN repair. It might be explained by the fact that the incidence of nerve injury after third molar surgery was relatively low in our population as reported in a previous local study [14]. The outcomes of the few cases of IAN repair might therefore help as a reference but is insufficient to draw a definitive conclusion of the outcome. This opens a door for future research on the long-term recovery pattern after IAN repair.

## Conclusion

Microsurgical treatment can provide a promising result in moderate to severe trigeminal nerve injury as a consequence of lower third molar surgery. The recovery pattern of IAN repair provides a reference but no conclusion is drawn due to the small sample size. Subjective and objective sensory improvement was achieved in the majority of these patients after microsurgery of LN. Most patients with pain as a symptom of the nerve injury recovered after the surgery. Subjective symptoms including numbness, taste sensation and speech were improved after LN repair. The impact on social life was found to be improved in most of the patients when compared to their pre-operative status. Improvement was noted in all three objective neurosensory tests at post-operative 12 months. Protective pain sensation as shown on the pain threshold test was restored in all the patients who had microsurgery of LN and has achieved 100% FSR by 6 months post-operatively.
